# Altered dopaminergic pathways and therapeutic effects of intranasal dopamine in two distinct mouse models of autism

**DOI:** 10.1186/s13041-020-00649-7

**Published:** 2020-08-10

**Authors:** Owen Y. Chao, Salil S. Pathak, Hao Zhang, Nathan Dunaway, Jay-Shake Li, Claudia Mattern, Susanne Nikolaus, Joseph P. Huston, Yi-Mei Yang

**Affiliations:** 1 Department of Biomedical Sciences, University of Minnesota Medical School, 1035 University Drive, Duluth, MN 55812 USA; 2 Department of Psychology, National Chung Cheng University, Minhsiung, Chiayi, Taiwan, Republic of China; 3 M et P Pharma AG, Emmetten, Switzerland; 4 Oceanographic Center, Nova Southeastern University, Fort Lauderdale, FL 33314 USA; 5 Clinic of Nuclear Medicine, Heinrich Heine University of Düsseldorf, Düsseldorf, Germany; 6 Center for Behavioral Neuroscience, Heinrich Heine University of Düsseldorf, Universitaetsstr. 1, 40225 Düsseldorf, Germany; 7 Department of Neuroscience, University of Minnesota, Minneapolis, MN 55455 USA

**Keywords:** Autism, Fragile X syndrome, BTBR, Fmr1, Striatum, Social behavior

## Abstract

The dopamine (DA) system has a profound impact on reward-motivated behavior and is critically involved in neurodevelopmental disorders, such as autism spectrum disorder (ASD). Although DA defects are found in autistic patients, it is not well defined how the DA pathways are altered in ASD and whether DA can be utilized as a potential therapeutic agent for ASD. To this end, we employed a phenotypic and a genetic ASD model, i.e., Black and Tan BRachyury T^+^Itpr3^tf^/J (BTBR) mice and Fragile X Mental Retardation 1 knockout (*Fmr1*-KO) mice, respectively. Immunostaining of tyrosine hydroxylase (TH) to mark dopaminergic neurons revealed an overall reduction in the TH expression in the substantia nigra, ventral tegmental area and dorsal striatum of BTBR mice, as compared to C57BL/6 J wild-type ones. In contrast, *Fmr1*-KO animals did not show such an alteration but displayed abnormal morphology of TH-positive axons in the striatum with higher “complexity” and lower “texture”. Both strains exhibited decreased expression of striatal dopamine transporter (DAT) and increased spatial coupling between vesicular glutamate transporter 1 (VGLUT1, a label for glutamatergic terminals) and TH signals, while GABAergic neurons quantified by glutamic acid decarboxylase 67 (GAD67) remained intact. Intranasal administration of DA rescued the deficits in non-selective attention, object-based attention and social approaching of BTBR mice, likely by enhancing the level of TH in the striatum. Application of intranasal DA to *Fmr1-*KO animals alleviated their impairment of social novelty, in association with reduced striatal TH protein. These results suggest that although the DA system is modified differently in the two ASD models, intranasal treatment with DA effectively rectifies their behavioral phenotypes, which may present a promising therapy for diverse types of ASD.

## Introduction

Autism spectrum disorder (ASD) is a prevailing neurodevelopmental disorder, primarily diagnosed by a core of symptoms including social impairments, communication difficulties, restricted interests and repetitive behaviors [1]. ASD patients often show cognitive and mental deficits comorbid with other neuropsychiatric disorders, such as attention-deficit/hyperactivity disorder (ADHD), anxiety, and bipolar disorder [2]. A system-level analysis of brain transcriptome has pointed out that the patterns of gene expression in schizophrenia, bipolar disorder and ASD significantly overlap, and that neurons/synapses are susceptible targets of polygenic modulations in all cases [3]. The common genetic variants and phenotypic traits among these disorders indicate shared neuropathology in the cell signaling pathways.

The dopamine (DA) system is an intriguing candidate. In the brain, tyrosine hydroxylase (TH) catalyzes the hydroxylation of tyrosine to L-DOPA, which is further converted to the modulatory neurotransmitter DA. DA binds to a large family of G-protein coupled receptors that are classified into two subgroups: D1-like (D1 & D5) and D2-like (D2-D4) receptors. Dopaminergic neurons primarily originate from substantia nigra pars compacta (SNc) and the ventral tegmental area (VTA) in the midbrain. Projections from SNc to the striatum (STR) form the nigrostriatal pathway that is important for controlling voluntary movement. Projections from VTA to the nucleus accumbens (NAc) and the frontal cortex make up the mesocorticolimbic pathway to regulate memory, reward, motivation and emotion [4]. Similar to ADHD [5], bipolar disorder [6] and schizophrenia [7], DA dysfunction is linked to ASD [8]. The STR and the frontal cortex that receive dopaminergic inputs are altered in human ASD [9–11] as well as in animal models with autistic-like behaviors induced by environmental factors [12]. In vivo imaging data demonstrate presynaptic alterations of DA synthesis and DA transporter (DAT) in the striatal and frontal cortical regions [13]. Interestingly, drugs involved in DA actions, such as risperidone, clozapine, haloperidol and methylphenidate, have yielded beneficial effects in ASD patients [14], although none of them acts selectively on the DA system.

To investigate how the dopaminergic pathways are modified and whether application of DA can have a therapeutic effect in ASD, we employed two distinct mouse models for ASD, i.e., Black and Tan BRachyury T^+^Itpr3^tf^/J (BTBR) and Fragile X Mental Retardation 1 knockout (*Fmr1*-KO) mice. The BTBR strain is a phenotypic model for idiopathic ASD, which exhibits impaired sociability, altered ultrasonic vocalization and increased self-grooming behaviors, simulating the main symptoms of human ASD [15]. We also found that BTBR mice display cognitive and emotional abnormalities akin to the psychiatric comorbidity of ASD [16, 17]. In addition, similar neuroanatomical changes between the BTBR model and ASD subpopulations are reported [18, 19]. As to the DA system, BTBR mice show reduced D2, but not D1, receptor-mediated neurotransmission [20]. On the other hand, *Fmr1*-KO mice are a genetically defined model for Fragile X syndrome (FXS) [21]. FXS is the result of transcriptional silencing of *Fmr1* gene and loss-of-function of its product, FMR protein (FMRP). Given that FXS is a leading inherited form of mental retardation and autism [22], *Fmr1*-KO animals are widely used for ASD-relevant studies. Characterizations of the *Fmr1*-KO mouse line have revealed a decreased number of SNc cells [23], compromised extracellular DA release [24] and disrupted D1 receptor-mediated synaptic transmission in the prefrontal cortex [25, 26].

In comparison with wild type (WT) control mice, we used biochemistry, immunohistochemistry and imaging methods to analyze dopaminergic, glutamatergic and GABAergic neurons in the DA pathways in BTBR and *Fmr1*-KO animals, with respective antibodies against TH, vesicular glutamate transporter 1 (VGLUT1) and glutamic acid decarboxylase 67 (GAD67). Fractal analysis of TH-positive axons in the STR was applied to reveal morphological changes of the dopaminergic projections and their spatial relationships with VGLUT1-immunoreactive nerve terminals. Moreover, we evaluated the effects of intranasal application of DA on the behavior and protein expression in BTBR and *Fmr1*-KO mice. Our results indicate that the DA system is altered differently yet intranasal treatment with DA improves the behavioral deficits in both mouse models, presenting a potential therapy for ASD.

## Methods

### Subjects

BTBR (stock # 002282), *Fmr1*-KO (stock # 003025) and C57BL/6 J (stock # 000664) mice were purchased from Jackson Laboratory (Bar Harbor, ME) and were housed in a facility accredited by the Association for the Assessment and Accreditation of Laboratory Animal Care. All mice were kept under a 12-h light-dark cycle (light on from 07:00 to 19:00) and reared 3–5 per cage with food and water ad libitum. Male and female mice were maintained in the same room. All procedures were approved by the Institutional Animal Care and Use Committee and the Institutional Biosafety Committee of University of Minnesota, in accordance with the National Institutes of Health guidelines. C57BL/6 J mice served as control because they are the most commonly adopted control for BTBR mice [27], and share the same genetic background with *Fmr1*-KO mice (https://www.jax.org/strain/003025). Unless specified, male mice (2–4 months old) were used for experiments due to the male-dominant prevalence of ASD [28].

### Immunohistochemistry

Mice (*n* = 3 mice/group for each set of experiments) were anaesthetized with ketamine (100 mg/kg, i.p.) and xylazine (10 mg/kg, i.p.), and transcardially perfused with phosphate-buffered saline (PBS, pH 7.4), followed by 4% paraformaldehyde. Brains were removed, post-fixed in 4% paraformaldehyde overnight at 4 °C, then immersed in 30% sucrose solution and stored at 4 °C until they sank. 50 μm-thick coronal sections were made on a microtome (Leica VT1200 S, Buffalo Grove, IL). They were maintained in 0.3% H_2_O_2_ solution for 10 min, rinsed with PBS and incubated in blocking solution containing 2% goat serum and 0.2% Triton-X 100 at 37 °C for 30 min. Sections were labelled with a rabbit TH antibody (1:800; Abcam ab112, Cambridge, MA) at 4 °C overnight. After being washed with 0.2% Triton-X 100 PBS solution, they were incubated in biotinylated anti-rabbit blocking solution for 2 h (1:200; Vector Lab. BA-1000, Burlingame, CA). They were then transferred to an ABC reagent (Vector Lab. PK-6100), stained with 3,3′-Diaminobenzidine (Vector Lab. SK-4100), mounted on slides and cover-slipped with Vectashield (Vector Lab. H-5000). To add fluorescence, after incubation in the primary antibody, sections were incubated in goat anti-rabbit Alexa Fluor 555 (1:1000; Invitrogen A21428, Waltham, MA). 3 h later, they were washed in PBS and cover-slipped with Vectashield (Vector Lab. H-1500). Immunostaining procedures for VGLUT1 and GAD67 were the same, except that primary antibodies guinea pig anti-VGLUT1 (1:5000; Millipore AB5905, St Louis, MO) and mouse anti-GAD67 (1:1000; Millipore MAB5406); and secondary antibodies goat anti-guinea pig Alexa 488 (1:1000; Invitrogen A11073) and goat anti-mouse Alexa 555 (1:1000; Invitrogen A32727) were used.

### Imaging analysis

Images were taken with a Zeiss LSM 710 confocal microscope with 20x and 63x oil immersion objectives or a Leica DMi8 light microscope with a 10x lens. Experimenters, who were blind to the design, analyzed the data using NIH ImageJ software (https://imagej.nih.gov/ij/). A region of interest (ROI) was selected based on a mouse brain atlas [29]. Areas of anti-TH, −VGLUT1 and -GAD67 signals were manually circled and summated with ImageJ. For each brain region, 2–3 images were sequentially taken with an inter-section interval of 200 μm. For each image, the intensity of ROI was subtracted from its background. The values of ROI intensity were then normalized to the average values of WT controls. Synaptic boutons were defined within the size of 0.2–3 μm^2^ of VGLUT1-positive signals. Co-localization of TH- and VGLUT1-positive boutons were identified with overlaps of the two fluorescent signals.

Fractal analysis of neuronal morphology is a validated methodology [30]. Striatal TH-positive axons were imaged with the 63x lens (6 Z-planes, 0.35 μm steps, field size: 30 × 30 μm^2^, pixel size: 1024 × 1024). Images were stacked to 2D and processed through deconvolution with ImageJ. A plugin FracLac (https://imagej.nih.gov/ij/plugins/fraclac/FLHelp/Installation.htm) with an implemented box counting method [31] was applied to calculate box-counting dimension (Db) and lacunarity. Db and lacunarity are fractal dimensional parameters used for quantifying complexity and inhomogeneity of digital spatial patterns, respectively.

### Western blotting

The striatum (*n* = 4–6 mice/group) was dissected bilaterally at 4 °C and stored at − 80 °C until processed. As described earlier [32], tissues were homogenized in ice-cold lysis buffer, centrifuged at 14,000 rpm for 10 min and the supernatants were collected for determination of protein concentration. Aliquots of protein (20–30 μg) were subjected to 10% SDS-PAGE and transferred to Immun-Blot PVDF Membrane (Bio-Rad, Hercules, CA). Membranes were incubated in 10% dry milk in PBST solution at room temperature for 1.5 h and then in primary antibodies at 4 °C overnight. Subsequently, they were washed with PBST for 30 min and incubated in secondary anti-mouse (1:5000; Abcam ab205719) and anti-rabbit (1:5000; Abcam ab205718) antibodies at room temperature for 1.5 h. Following a rinse with PBST for 30 min, they were reacted with enhanced chemiluminescent reagent (GE Healthcare Life Sciences, Buckinghamshire, UK) and imaged with Odyssey Fc Imaging System (LI-COR Biosciences, Lincoln, NE). Blot intensity was quantified using ImageJ and normalized to β-actin.

Primary antibodies included rabbit anti-TH (1:500; Abcam ab112), guinea pig anti-VGLUT1 (1:1000; Millipore AB5905), mouse anti-GAD67 (1:5000; Millipore MAB5406), mouse anti-DAT (1:1000; NovusBio mAb16, Littleton, CO) and mouse anti-β-actin (1:5000; Sigma-Aldrich A5441, St. Louis, MO).

### Dopamine administration

Mice were handled once a day for at least three consecutive days before drug administration. Dopamine hydrochloride (Sigma-Aldrich H8502) was suspended in a viscous castor oil-based formulation (MetP Pharma, Emmetten, Switzerland). It was freshly prepared in a dose of 3 mg/kg in a volume of 10 μl and kept on ice with protection from light. DA or vehicle suspension was applied 5 μl per nostril for ~ 30 s with a pipette (Eppendorf North America, Hauppauge, NY). 10 min after administration, animals underwent elevated plus maze, open field or object-based attention tests. Because of their respective deficit in social approaching or social novelty [16, 33], in the three-chamber test, BTBR mice received DA before the habituation trial, whereas *Fmr1*-KO mice received DA before the sociability trial. As BTBR mice have intact recognition of social novelty [16, 17], the social novelty trial was excluded here to avoid excessive administration of DA in the same subjects within a short time. The dosage and timing of DA administration were based on a previous study in mice [34] .

### Behavioral testing

BTBR mice (*n* = 12; 8 males and 4 females) were tested using a within-subject design to minimize individual differences. Choice of sex was based on our earlier report that both male and female BTBR mice show autistic-like behaviors [17]. Half of the animals were treated with DA and the other half with vehicle. The behavioral tests were conducted in the order: elevated plus maze, open field, object-based attention and three-chamber social test, with an inter-test interval of 24–48 h. One week after the final test, the previous DA group received vehicle and vice versa, followed by the same behavioral testing.

*Fmr1*-KO mice were randomly divided into vehicle (*n* = 7 males) or DA (*n* = 9 males) group and tested using a between-subject design. Choice of sex was based on the location of *Fmr1* gene on the X chromosome with male-dominant occurrence [21]. The order of behavioral tests and the inter-test interval were the same as for BTBR mice. Each *Fmr1*-KO animal was exposed to each test only once.

Behavioral testing was done in a quiet room (< 40 dB) during 10:00–16:00. LED light provided dim illumination (~ 50 lx). Male mice were tested before female ones. Apparatus was cleaned with 70% ethanol between animals to remove pheromones. A camera was connected to a computer for tracking animals and recording videos (Stoelting ANY-maze, Wood Dale, IL).

#### Elevated plus maze

This test was used to assess anxiety-like behaviors [35]. The maze has two open arms (30 × 5 cm), two closed arms (30 × 5 cm) and a central platform (5 × 5 cm) and is elevated at 30 cm height. The subject was placed on the central platform facing the open arms and given 5 min to travel freely. Entries to and time spent in the center, open and closed arms, and head-dips, were counted.

#### Open field

This test was used to assess locomotion and exploration behaviors. The subject was placed in a polyvinyl chloride box (40 × 40 × 30 cm) for 15 min. Distance traveled, thigmotaxis (distance travelled along the walls), duration of grooming, time spent in the center (virtual central square 13.3 × 13.3 cm) and counts/duration of rearing were analyzed for 15 min in three 5-min bins. Rearing is a sign for non-selective attention [36]. Self-grooming is a measure of repetitive behavior [37]. Thigmotaxis is an index for sensorimotor function and/or anxiety [38, 39]. Time spent in the center indicates the anxiety level [40]. One BTBR mouse was excluded from the analysis due to lack of rearing.

#### Object-based attention test

This test was used to assess attention-associated processes and/or short-term working memory [16]. Objects of different materials (plastic or glass), textures (smooth or rough), sizes (diameter 7–9 cm, height 14–17 cm) and shapes (column or irregular) were placed in the open field. Objects weighed enough to prevent being displaced by the animal. Assignment of the objects was counterbalanced to minimize a potential bias for their identity or location. Prior to testing, the animal was habituated to the open field without objects. The actual test consisted of a learning trial (5 min) and a test trial (5 min) without time delay in between. In the learning trial, the animal was introduced to the field which contained two distinct objects. In the test trial, the objects were replaced by a new one and a copy of either of the explored ones in the same location. During the replacement, the animal remained inside the arena. Object exploration was defined as physical contacts with the objects by the animal’s nose, head and forepaws, but not by the body or tail. Climbing or sitting aside the objects was not included. Animals that explored the objects for < 10 s in either trial were excluded from the analysis. Here, the index = [time spent on the novel object - time spent on the old object] / total time spent on both objects, with a positive value representing intact performance.

#### Three-chamber sociability and social novelty test

This test was used to assess sociable behavior and recognition of social novelty [41], in a polyvinyl chloride apparatus composed of three chambers (20 × 40 × 30 cm each) with passages (5 × 5 cm) dividing the chambers. The test included three sessions: habituation, sociability and social novelty (9 min each). In the habituation trial, the subject was placed into the middle chamber and allowed to freely explore the whole apparatus. In the sociability trial, a gender- and age-matched WT mouse that had never been contacted by the subject was put underneath a metal grid cup (diameter 10 cm, height 12 cm) in one of the side chambers. Another identical cup was put in the opposite side chamber. The locations for placing the stranger mouse and the empty cup were counterbalanced between subjects. In the social novelty trial, another stranger mouse was placed underneath the previously empty cup. The same strangers were used between subjects. Physical contacts around the cups by the subject’s nose, head and forelimbs were defined as explorative behaviors. Sociable index = [time for exploring the stranger mouse - time for exploring the empty cup] / total exploration time. Social novelty index = [time for exploring the novel mouse - time for exploring the familiar mouse] / total exploration time. Positive values represent intact sociability and social novelty preference.

### Statistics

Repeated two-way ANOVAs with “within-subject” factors (treatment, interval or object) were used in the analyses of BTBR behaviors. Mixed two-way ANOVAs with a “between-subject” factor (group) and a “within-subject” factor (interval or object) were used in the analyses of *Fmr1*-KO behaviors. One-way ANOVAs, paired *t*-tests and one-sample *t*-tests were applied when appropriate. Independent *t*-tests (if allowed by results of one-way ANOVAs) or Mann-Whitney U tests (in case of lack of homogeneity or normality of variance) were used for analyzing immunohistochemistry and Western blotting data. Data were expressed as mean ± standard error of mean (SEM). Statistical significance was set as *p* < 0.05. All tests were two-tailed tests. For imaging analyses, *n* denoted the number of samples from 3 mice per group. Otherwise, *n* represented the number of mice per group. Sample sizes were determined on the basis of previous studies using similar experimental protocols [16, 17, 33].

## Results

### Differential alterations of the DA system in BTBR and *Fmr1*-KO mice

**Fig. 1:**
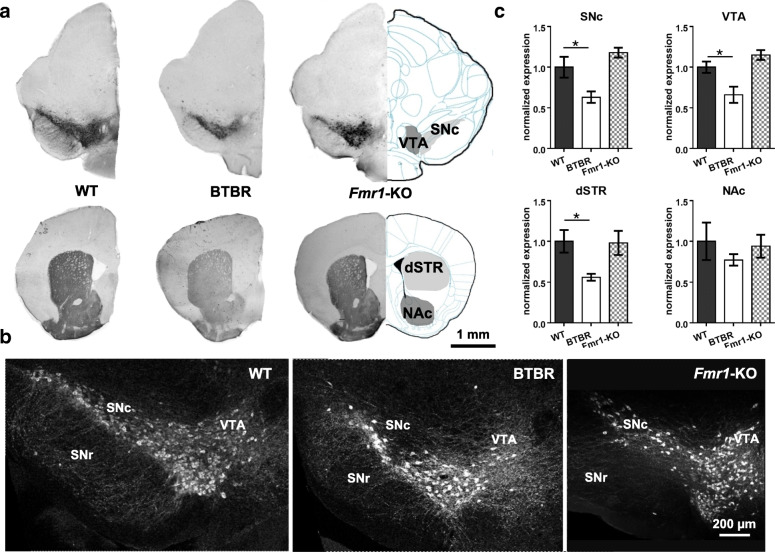
Immunohistochemical analyses of TH expression in WT, BTBR and *Fmr1*-KO mice. **a** Representative diagrams and images of anti-TH staining in substantia nigra pars compacta (SNc), ventral tegmental area (VTA), dorsal striatum (dSTR) and nucleus accumbens (NAc). **b** Examples of confocal images (20x) of dopaminergic neurons in WT, BTBR and *Fmr1*-KO mice. **c** Fluorescence intensity of anti-TH staining in the region of interest (ROI) was measured in an identical microscopic setting and normalized to the WT animals. The BTBR brain exhibited decreased TH-positive expression in SNc, VTA and dSTR, while the *Fmr1-* KO brain did not. TH: tyrosine hydroxylase; SNr: substantia nigra pars reticulata. *n* = 6–7 samples/group. *p < 0.05, compared to WT

To investigate whether and how the DA pathways are modified in the ASD models, we labelled dopaminergic neurons with an anti-TH antibody in brain sections taken from age-, sex- and background matched WT, BTBR and *Fmr1*-KO mice. TH is a rate-limiting enzyme that converts tyrosine to DA precursor L-DOPA. We first examined the origins of the DA system, i.e. the SNc and VTA in the midbrain, and their major projections to the dorsal striatum (dSTR) and NAc via the respective nigrostriatal and mesocorticolimbic pathways [4]. Diagrams in Fig. 1a depict the distribution of the soma and axons of dopaminergic neurons identified by TH labelling in the four nuclei. Higher-magnification images illustrate the specific locations of these neurons within the SNc and VTA (Fig. 1b). By measuring the intensities of anti-TH staining and normalizing them to the average values of the WT group, we found that TH expression in the BTBR brains was significantly reduced in SNc (U = 6, Z = 2.146, *p* = 0.032), VTA (U = 7, Z = 2.003, *p* = 0.045), and dSTR (U = 6, Z = 2.143, *p* = 0.032; Mann-Whitney U test) (Fig. 1c). In contrast, no differences in TH expression were observed in the brain areas between *Fmr1*-KO and WT mice (*p* > 0.05; Mann-Whitney U test).

**Fig. 2:**
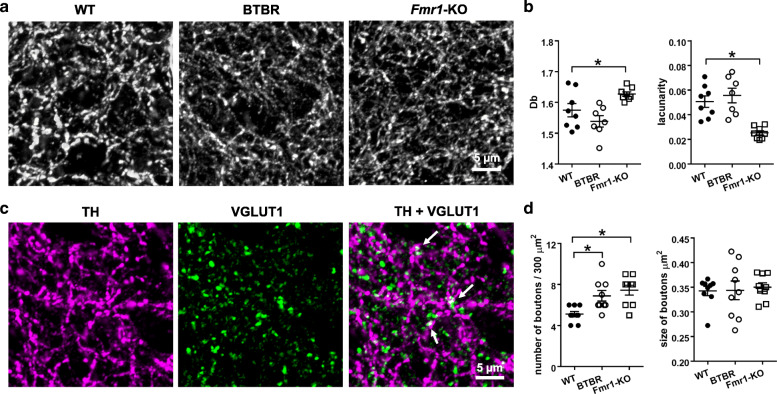
Fractal analyses of TH-positive axons and measurements of co-labelled TH and VGLUT1 signals in the WT, BTBR and *Fmr1*-KO striatum. **a** Representative images of anti-TH staining in the dorsal striatum, taken by a confocal microscope with a 63x lens, forming a 30 × 30 μm field with 6 z-stacks of 0.35 μm steps. **b** Fractal dimensions Db of TH-positive axons in the *Fmr1*-KO striatum was higher, while the value of lacunarity was lower, than those in the WT striatum. **c** Representative diagrams of anti-TH (magenta), anti-VGLUT1 (green) and their merged image in the WT striatum. Merged dots (white), pointed by arrows, indicate boutons with adjacent TH and VGLUT1 labelling. **d** Boutons with the co-labelled signals were counted and their size was measured. The size of the boutons was similar between groups, whereas the number of the boutons was more in BTBR and *Fmr1*-KO mice than that in WT controls. *n* = 7–9 samples/group. *p < 0.05, compared to WT

As the STR (mostly dSTR) is a key substrate of the DA outputs and crucially involved in ASD [11], we subsequently focused on the dopaminergic innervations in this region. We noticed a different pattern of TH-positive axons in *Fmr1*-KO mice as compared to WT and BTBR animals (Fig. 2a). Quantitative assessments of fractal dimensions [30] unraveled significant “group” effects in Db (F_2,20_ = 6.887, *p* = 0.005) and lacunarity (F_2,20_ = 14.4, *p* < 0.001). The *Fmr1*-KO group had higher Db (t_14_ = − 2.304, *p* = 0.037; increased “complexity”) and lower lacunarity (t_14_ = 5.126, *p* < 0.001; decreased “texture”), while BTBR mice displayed no such differences from the WT cohort (*p* > 0.05) (Fig. 2b).

Beside the dopaminergic afferents, the STR receive glutamatergic and GABAergic inputs, and their interactions are essential for the functionality of the basal ganglia. The main excitatory projections come from the cortex and thalamus, which end with terminal boutons that are immunoreactive to VGLUT1 and VGLUT2, respectively [42, 43]. The two populations of nerve terminals are comparable in the amount and in a similar spatial relation with dopaminergic axons [44]. The inhibitory synapses largely arise from different types of interneurons in the STR and medium spiny neurons in the “direct” (striatonigral) and “indirect” (striatopallidal) pathways [45]. Staining GABAergic neurons with an anti-GAD67 antibody showed no difference in the intensity of GAD67 labelling among the three groups (WT: 1.00 + 0.15, BTBR: 0.97 + 0.09, *Fmr1*-KO: 1.31 + 0.20; *p* > 0.05). Likewise, using VGLUT1 as a marker for glutamatergic synapses, we did not detect any significant variance in the overall expression of VGLUT1 (WT: 1.00 + 0.11, BTBR: 1.15 + 0.12, *Fmr1*-KO: 1.35 + 0.24; *p* > 0.05). However, in the analysis of co-labelled VGLUT1 and TH signals, we uncovered an increased number of VGLUT1-possitive boutons co-localized with the TH-positive axons/synapses in the BTBR (t_16_ = − 3.094, *p* = 0.007) and *Fmr1*-KO (t_16_ = − 4.309, *p* = 0.001) STR, compared with the WT brain (Fig. 2c & d). The size of these boutons did not differ (*p* > 0.05).

**Fig. 3:**
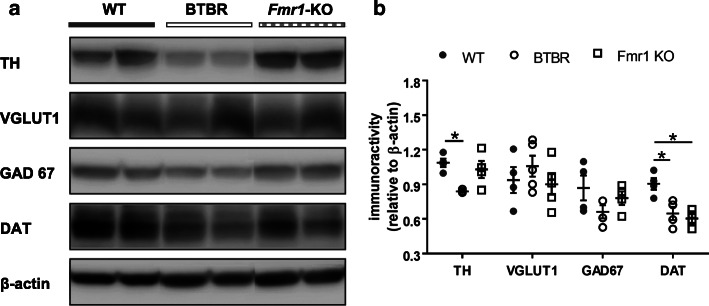
Western blotting of TH, VGLUT1, GAD67 and DAT in the striatum. **a** Examples of Western blots of striatal lysates from WT, BTBR and *Fmr1*-KO mice. Images of protein bands were aligned for comparison. **b** Blot intensity was normalized to an internal standard β-actin. Decreased TH and DAT levels were found in BTBR mice, while *Fmr1*-KO animals only showed reduced DAT expression, as compared to the WT group. Relative quantities of other proteins were comparable among groups. VGLUT1: vesicular glutamate transporter 1; GAD67: glutamate decarboxylase 67; DAT: dopamine transporter; TH: tyrosine hydroxylase. *n* = 4–5 mice/group. *p < 0.05, compared to WT

Next, we performed Western blotting from striatal homogenates to estimate the quantities of TH, VGLUT1 and GAD67 proteins. In agreement with the immunohistochemical findings (Fig. 1), the total amount of TH in the STR of BTBR mice was substantially lower than that in the WT brains (U = 0, Z = -2.309, *p* = 0.021), while VGLUT1 and GAD67 were unaltered (*p* > 0.05) (Fig. 3a & b). No differences were found in the amount of TH, VGLUT1 and GAD67 between *Fmr1*-KO and WT groups (*p* > 0.05). Furthermore, we measured DAT, an essential presynaptic protein that modulates DA homeostasis by the reuptake of DA. De novo mutation of DAT gene is a risk factor for ASD [8]. Interestingly, the DAT levels dropped in both BTBR (U = 0, Z = -2.309, *p* = 0.021) and *Fmr1*-KO (U = 0, Z = -2.309, *p* = 0.021) mice, as compared to the WT controls. These changes in the protein expression were confirmed by normalized quantities of TH, VGLUT1, GAD67 and DAT in the BTBR and *Fmr1*-KO STR relative to the WT cohorts (Table 1).

**Table 1:** Results of Western blots normalized to wild-type (WT) or vehicle (VEH)-treated groups. *TH* tyrosine hydroxylase, *VGLUT1* vesicular glutamate transporter 1, *GAD67* glutamic acid decarboxylase 67, *DAT* dopamine transporter, *DA* dopamine, *KO* knockout, *N.A.* not applicable. *p < 0.05, compared to WT or VEH groups; Mann-Whitney test. Values are shown as mean ± SEM

	TH	VGLUT1	GAD67	DAT
WT	1.00 ± 0.05	1.00 ± 0.18	1.00 ± 0.10	1.00 ± 0.04
BTBR	0.80 ± 0.04*	1.20 ± 0.05	0.94 ± 0.08	0.72 ± 0.05*
*Fmr1*-KO	1.20 ± 0.17	1.05 ± 0.12	1.10 ± 0.08	0.64 ± 0.05*
BTBR + VEH	1.00 ± 0.05	N.A.	N.A.	1.00 ± 0.05
BTBR + DA	1.20 ± 0.07*	N.A.	N.A.	1.00 ± 0.06
*Fmr1*-KO + VEH	1.00 ± 0.05	N.A.	N.A.	1.00 ± 0.05
*Fmr1*-KO + DA	0.71 ± 0.07*	N.A.	N.A.	1.15 ± 0.23

Taken together, BTBR mice exhibited a global reduction of TH expression in the cell body and axon projections of dopaminergic neurons in multiple nuclei, suggesting severe detriments along the DA pathways. *Fmr1*-KO animals did not have such alterations yet showed abnormal morphology of TH-positive axons in the STR. Both strains evidenced more VGLUT1 in close proximity to the TH signals, indicating an altered regulation of the excitatory inputs by DA. Lastly, the decreased amount of striatal DAT implies deficient DA reuptakes in the two models.

### Effects of intranasal DA on striatal protein expression in BTBR and *Fmr1*-KO mice

**Fig. 4:**
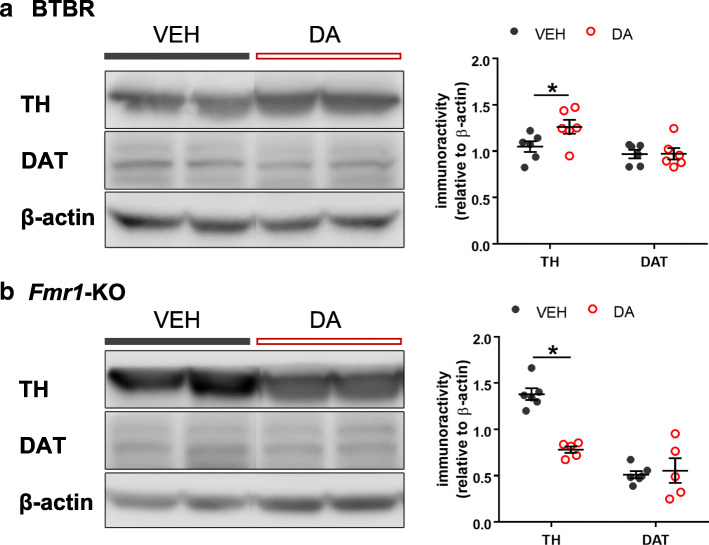
Immunoblotting of TH and DAT in the striatum after intranasal delivery of vehicle (VEH) or dopamine (DA). **a** Western blots of striatal lysates from BTBR mice treated with VEH or DA. Increased TH expression was found after DA administration. **b** Western blots of striatal lysates from *Fmr1*-KO mice treated with VEH or DA. Reduced TH level was noticed after DA application. In both cases, DAT protein was unaltered by intranasal DA. TH: tyrosine hydroxylase; DAT: dopamine transporter. *n* = 5–6 mice/group. *p < 0.05, compared to VEH

Knowing the DA system was dysregulated in the ASD models (Figs. 1, 2, 3), we hypothesized that application of DA might rectify their phenotypes. Because DA cannot pass the blood-brain-barrier due to its polar properties, we administrated DA via the nasal passage [46]. BTBR and *Fmr1*-KO mice were randomly assigned to vehicle and DA treatments, separately. They were sacrificed 15 min after intranasal administration of either reagent. We quantified the proteins that were altered in their STR with immunoblotting. Compared to the vehicle groups, DA application increased the expression of TH in the BTBR STR (U = 4, Z = 2.242, *p* = 0.025) (Fig. 4A & Table 1), but decreased it in the *Fmr1*-KO STR (U = 0, Z = 2.739, *p* = 0.006) (Fig. 4b & Table 1). Given the basal level of TH was lower in BTBR than that in WT mice (Fig. 3), this result implicates that intranasal DA may help rectify the deficiency in the BTBR striatal circuit. In the *Fmr1*-KO model, the decreased amount of TH following DA delivery may relate to a modulation of FMRP-mediated DA signaling (see Discussion). Although DAT was reduced in both of the ASD strains (Fig. 3), no significant changes were found after DA administration (*p* > 0.05, Fig. 4 & Table 1). Nevertheless, the susceptibility of TH protein to intranasal application of DA rationalizes the utility of DA for behavioral rescues.

### Intranasal delivery of DA alleviates the deficits in non-selective attention, object-based attention and sociability of BTBR mice

**Fig. 5:**
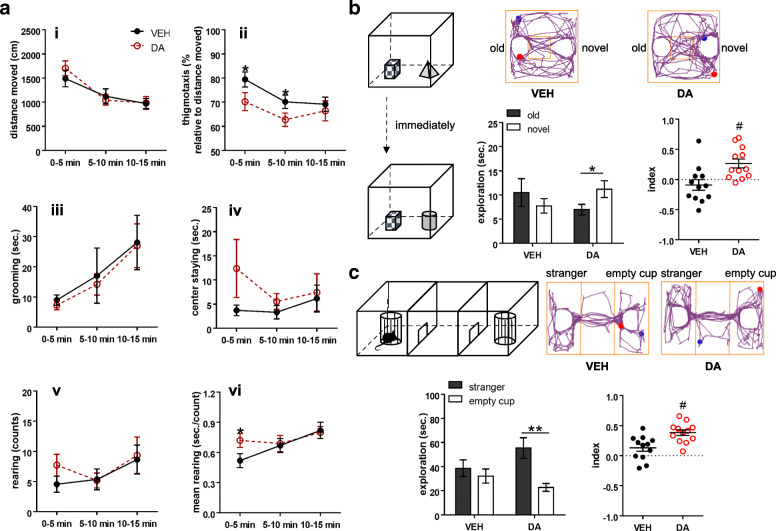
Behavioral effects of intranasal dopamine on BTBR mice. **a** Behaviors measured in the open field test. The dopamine (DA)-treated group showed comparable distance travelled (i), self-grooming (iii), center staying (iv), and counts of rearing (v) to the vehicle (VEH)-treated group, except for a lower percentage of thigmotaxis at the time intervals 0–5 and 5–10 min (ii) and a longer duration of rearing in the first 5 min (vi). **b** Behaviors assessed in the object-based attention test. An example of movement tracking of a VEH- or DA-treated animal is illustrated. Blue and red dots indicate the start and end points of a test trial, respectively. Unlike the VEH-treated animals, the DA-treated ones exhibited the preference toward the novel object, and thus, showing positive values of the index. Index = [time spent on the novel object - time spent on the old object] / total exploration time. **c** Behaviors evaluated in the three-chamber social test. An example of movement tracking of a VEH- or DA-treated animal is presented. Blue and red dots indicate the start and end points of a sociability trial, respectively. The VEH-treated animals explored the stranger mouse and the empty cup equally, while the DA-treated ones showed the preference to the stranger mouse and had positive values of the sociable index. Index = [time for exploring the stranger mouse - time for exploring the empty cup] / total exploration time. *n* = 12 mice/group. *p < 0.05, **p < 0.01, compared to the respective values. #*p* < 0.05, compared to 0 by one sample *t*-test

We performed behavioral assays following intranasal delivery of vehicle or DA to BTBR mice. In the open field test, we quantified the parameters in three intervals by taking into account confounding factors (anxiety/habituation) that could influence rodent locomotor activity [47]. Analysis of distance travelled with a repeated two-way ANOVA revealed a significant effect of “interval” (F_2, 12_ = 30.519, *p* < 0.001), but not of “treatment” or “treatment x interval” (*p* > 0.05; Fig. 5a i). Since the “interval” effect was present, one-way ANOVAs with the within-subject factor “treatment” were applied separately for the periods of 0–5, 5–10, 10–15 min. No “treatment” differences in the travelling distance were detected at any time intervals (*p* > 0.05). In the analysis of thigmotaxis, we found a significant “interval” effect (F_2, 12_ = 7.186, *p* = 0.004), but not “treatment” or “treatment x interval” effect (*p* > 0.05; Fig. 5a ii). Subsequent examination for each interval uncovered that DA treatment reduced thigmotactic behaviors of BTBR mice in the time bins of 0–5 min (F_1, 11_ = 6.359, *p* = 0.028) and 5–10 min (F_1, 11_ = 5.693, *p* = 0.036). While thigmotaxis is viewed as an index for sensorimotor function and anxiety [38, 39], the reduction of thigmotactic behaviors in the DA-treated animals cannot be readily explained by less anxiety because DA did not affect other parameters that reflect the anxiety level, including time spent in the center of the open field and performance on the elevated plus maze (see below). An alternative possibility is that DA may help control excessive thigmotaxic behaviors of BTBR mice [16], by refining their sensory-motor integration. Analysis of self-grooming showed a significant effect of “interval” (F_2, 22_ = 8.446, *p* = 0.002), but not of “treatment” or “treatment x interval” (*p* > 0.05; Fig. 5a iii). In each interval, no “treatment” difference was detected (*p* > 0.05). As to other assessments on time spent in the center and counts of rearing, no effects of “treatment”, “interval” or their interaction were observed (*p* > 0.05; Fig. 5a iv & v). By contrast, while calculating the average duration of rearing, an indicator for non-selective attention [36], we found a significant effect of “treatment x interval” (F_2, 14_ = 21.463, *p* < 0.001), but not of “treatment” or “interval” (*p* > 0.05; Fig. 5a vi). Essentially, the animals treated with DA spent more time on rearing than those receiving the vehicle in the first 5 min (F_1, 10_ = 5.146, *p* = 0.047) but not in other intervals (*p* > 0.05). As BTBR mice show non-selective attention deficits [16], we suggest that intranasal administration of DA improves their non-selective attentional processing without affecting their general locomotion or exploratory activity.

In the object-based attention test, there was no difference in the total time of object exploration between the DA and vehicle-treated groups in either the learning or the test session (*p* > 0.05; Fig. 5b & Table 2). Yet, analysis of the test trial unfolded a significant effect of “treatment x object” (F_1, 11_ = 7.73, *p* = 0.018), but not of “treatment” or “object” (*p* > 0.05). Paired *t*-tests were then used to compare the exploration time for the old versus the novel object within each treatment. The vehicle-treated BTBR mice explored both objects indiscriminately (*p* > 0.05), consistent with our previous report on their attention/memory deficiency [16]. In contrast, DA-treated animals preferred the novel to the old object (t_11_ = − 2.511, *p* = 0.029), giving a higher cognitive index (t_11_ = 3.588, *p* = 0.004). The results imply that DA enhances object-based attention and/or short-term memory of the BTBR model.

**Table 2:** Total exploration time (seconds) in the object-based attention test (OBAT) and three-chamber social test. *VEH* vehicle, *DA* dopamine, *N.A.* not applicable. Values are shown as mean ± SEM

	BTBR		*Fmr1*-KO	
OBAT	VEH	DA	VEH	DA
learning	21.51 ± 2.29	18.27 ± 1.22	24.86 ± 5.49	26.56 ± 2.19
test	18.16 ± 2.37	18.54 ± 3.04	17.62 ± 4.66	21.58 ± 2.93
Three-chamber				
sociability	70.88 ± 12.28	78.25 ± 10.92	62.00 ± 6.10	62.36 ± 8.25
social novelty	N.A.	N.A.	47.87 ± 6.42	59.18 ± 4.87

In the three-chamber social test, the total exploration time was comparable between the treatments (*p* > 0.05; Table 2). Significant effects of “treatment x object” (F_1, 10_ = 20.541, *p* = 0.001) and “object” (F_1, 10_ = 17.834, *p* = 0.002), but not of “treatment” (*p* > 0.05), were found in the sociability trial. Specifically, the DA-treated animals explored the stranger noticeably more than the empty cup (t_10_ = 4.901, *p* = 0.001; paired *t*-test; Fig. 5c), while the vehicle-treated mice did not (*p* > 0.05). Accordingly, the DA, but not the vehicle, treatment rendered a positive sociability index (t_10_ = 8.055, *p* < 0.001; one-sample *t*-test). Considering the characteristics of BTBR mice in their reduced sociability [15, 16], this result indicates a beneficial action of intranasal DA on their social impairments. As the BTBR strain has intact social novelty [15, 16], we did not continue into the social novelty trial to avoid excessive administration of DA in the same subjects within a short time.

The elevated plus maze test showed no differences in the total distance travelled, entries to and time spent in the center, open and closed arms, and counts of head-dips between the DA and vehicle treatments (*p* > 0.05; Table 3). This suggests that DA does not amend the high non-social anxiety associated with the BTBR animals [16].

**Table 3:** Behavioral assessments in the elevated plus maze test. *VEH* vehicle, *DA* dopamine. Values are shown as mean ± SEM

	BTBR		*Fmr1*-KO	
	VEH	DA	VEH	DA
distance (cm)	816.07 ± 57.81	965.20 ± 112.64	1194.93 ± 126.64	1091.41 ± 76.38
entry (counts)				
center	34.58 ± 3.06	36.42 ± 4.18	37.29 ± 2.29	36.67 ± 3.02
open	13.08 ± 1.56	12.92 ± 1.61	14.29 ± 1.91	15.22 ± 1.98
closed	22.92 ± 1.64	25.50 ± 3.02	26.71 ± 1.66	25.00 ± 1.59
time spent (seconds)				
center	48.88 ± 6.77	43.01 ± 4.92	71.37 ± 4.80	68.18 ± 7.86
open	32.78 ± 6.29	37.67 ± 4.99	52.89 ± 7.88	52.91 ± 9.39
closed	218.33 ± 11.00	219.32 ± 9.15	175.71 ± 8.19	178.90 ± 10.16
head-dips (seconds)	7.19 ± 2.32	7.74 ± 1.10	24.83 ± 2.54	30.47 ± 4.90

Biological variables, such as sex difference, could contribute to the above observations as both male (*n* = 8) and female (*n* = 4) BTBR mice were included. In the three-chamber social test, for example, conspecific interactions between male subjects and male strangers may have a different innate quality from those among females due to inter-male territoriality and aggression. To test the possibility, we reanalyzed the data by including the males only and found similar results from intranasal DA administration (Table 4), reinforcing the robust effects of DA on the BTBR behaviors.

**Table 4:** Behaviors of male BTBR mice treated with vehicle (VEH) or dopamine (DA). *p < 0.05, paired *t*-tests compared to VEH. #*p* < 0.05, paired *t*-tests compared to the empty cup exploration within the same group. †*p* < 0.05, one-sample *t*-tests compared to 0. Values are shown as mean ± SEM. Note: in the object-based attention test, although the preference for the novel over old object was moderate (*p* = 0.063), the object-recognition index of the DA-treated group remained positive

Elevated plus maze	VEH	DA
distance (cm)	863.63 ± 78.81	1036.18 ± 114.71
center entry (counts)	36.00 ± 4.41	38.75 ± 4.34
open-arms entry (counts)	14.38 ± 2.17	13.75 ± 2.02
closed-arms entry (counts)	23.38 ± 2.01	27.00 ± 2.56 *
center time (seconds)	51.05 ± 9.31	46.60 ± 5.74
open-arms time (seconds)	42.10 ± 7.07	42.23 ± 6.67
closed-arms time (seconds)	206.85 ± 14.32	211.19 ± 11.35
head-dips (seconds)	8.28 ± 3.02	8.85 ± 1.11
Open field		
distance 0–5 min (cm)	1451.73 ± 175.70	1831.94 ± 122.04
distance 5–10 min (cm)	1137.80 ± 233.01	1184.25 ± 106.78
distance 10–15 min (cm)	980.48 ± 158.01	1148.89 ± 155.66
thigmotaxis 0–5 min (% by distance)	76.99 ± 3.95	64.68 ± 4.33 *
thigmotaxis 5–10 min (% by distance)	66.20 ± 2.98	58.51 ± 2.97
thigmotaxis 10–15 min (% by distance)	67.12 ± 4.13	63.13 ± 5.42
grooming 0–5 min (seconds)	9.33 ± 2.51	6.43 ± 1.85
grooming 5–10 min (seconds)	21.09 ± 13.57	17.90 ± 4.83
grooming 10–15 min (seconds)	31.64 ± 12.88	30.43 ± 10.10
center time 0–5 min (seconds)	4.00 ± 1.60	7.94 ± 2.99
center time 5–10 min (seconds)	4.45 ± 2.00	7.46 ± 2.18
center time 10–15 min (seconds)	8.04 ± 4.04	10.13 ± 5.61
rearing 0–5 min (counts)	6.00 ± 1.85	8.13 ± 1.67
rearing 5–10 min (counts)	7.00 ± 2.54	6.00 ± 1.41
rearing 10–15 min (counts)	10.71 ± 3.61	11.88 ± 3.77
rearing 0–5 min (seconds/count)	0.61 ± 0.08	0.77 ± 0.07
rearing 5–10 min (seconds/count)	0.65 ± 0.10	0.69 ± 0.10
rearing 10–15 min (seconds/count)	0.74 ± 0.15	0.81 ± 0.08
Object-based attention		
total exploration (seconds)_learning	22.06 ± 3.40	20.40 ± 1.12
old object (seconds)_test	10.18 ± 2.78	6.15 ± 1.11
novel object (seconds)_test	8.61 ± 1.54	13.90 ± 3.77
index	−0.05 ± 0.12	0.31 ± 0.10 †
total exploration (seconds)_test	18.79 ± 3.24	20.05 ± 4.31
Three-chamber		
stranger exploration (seconds)	45.90 ± 8.23	60.69 ± 9.07 #
empty cup exploration (seconds)	37.43 ± 7.21	25.88 ± 3.81
index	0.12 ± 0.06	0.39 ± 0.06 †
total exploration (seconds)	83.33 ± 14.64	86.56 ± 11.81

### Intranasal application of DA to *Fmr1*-KO mice rectifies their defects in object-based attention and social novelty preference

**Fig. 6:**
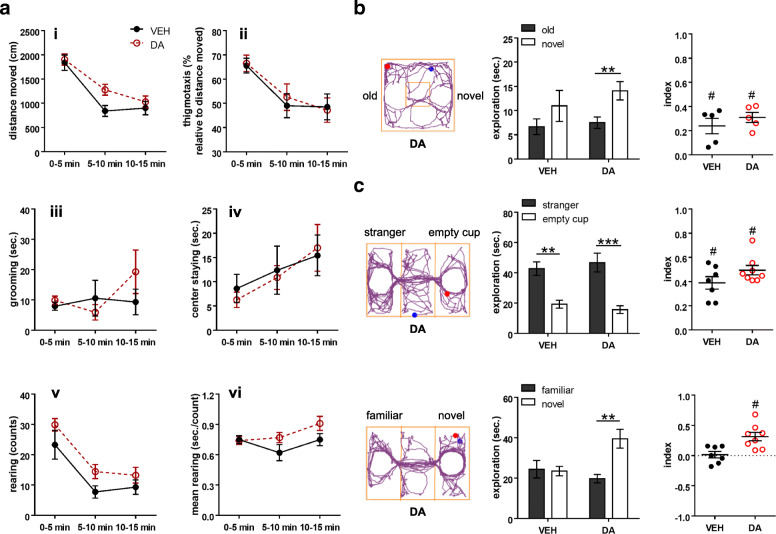
Behavioral effects of intranasal dopamine on *Fmr1-*KO mice. **a** Behaviors measured in the open field test. The dopamine (DA)-treated group showed comparable distance travelled (i), thigmotaxis (ii), self-grooming (iii), center staying (iv), counts of rearing (v), and average duration of rearing (vi) to the vehicle (VEH)-treated group. **b** Behaviors assessed in the object-based attention test. An example of movement tracking of a DA-treated animal is shown. Blue and red dots indicate the start and end points of a test trial, respectively. The DA-treated animals exhibited the preference toward the novel object, although both groups had positive values of the index. Index = [time spent on the novel object - time spent on the old object] / total exploration time. **c** Behaviors evaluated in the three-chamber social test. Examples of movement tracking of a DA-treated animal in a sociability and a social novelty trial are displayed. Blue and red dots indicate the start and end points of each trial, respectively. In the sociability trial (upper panels), both groups displayed social preference toward the stranger mouse and had positive values of the sociable index. Index = [time for exploring the stranger mouse - time for exploring the empty cup] / total exploration time. In the social novelty trial (bottom panels), the DA-treated group explored the novel stranger more than the familiar one, whereas the VEH-treated group did not. The DA-treated group thus had higher values of the social novelty index. Index = [time for exploring the novel mouse - time for exploring the familiar mouse] / total exploration time. *n* = 7–9 mice/group (2–4 mice were excluded in the object-based attention test due to < 10 s exploration). **p < 0.01, ***p < 0.001, compared to the respective values. #*p* < 0.05, compared to 0 by one sample *t*-test

We executed the same behavioral testing in *Fmr1*-KO mice after vehicle or DA treatments. In the open field test, a significant effect of “interval” (F_2,28_ = 62.865, *p* < 0.001; mixed two-way ANOVAs), but not of “group” or “group x interval” (*p* > 0.05), was detected in the analysis of distance travelled (Fig. 6a). Subsequent one-way ANOVAs showed no group difference in the travelling distance at any given time interval (*p* > 0.05). As for thigmotaxis behavior, there was a significant effect of “interval” (F_2,28_ = 100.306, *p* < 0.001), but not of “group” or “group x interval”. No group differences were found in the three intervals (*p* > 0.05). As for the time spent in the center, there was a significant “interval” (F_2,28_ = 6.895, *p* = 0.004), but not “group” or “group x interval”, effect (*p* > 0.05). No group differences were found at any intervals (*p* > 0.05). Assessments on self-grooming and counts and duration of rearing indicated no significant effects of “group”, “interval” or their interaction (*p* > 0.05).

In the object-based attention test, there were no group differences in the total time of object exploration throughout the learning and test sessions (*p* > 0.05; Table 2). In the test trial, there was a significant effect of “object” (F_1,8_ = 22.516, *p* = 0.001), but not of “group” or “group x object” (*p* > 0.05). The DA group explored the novel object more than the old one (t_4_ = − 5.423, *p* = 0.006), whereas the vehicle group did not (*p* > 0.05; Fig. 6b). Both cohorts had positive object-based attention scores (t_4_ = 7.378, *p* = 0.002 for DA; t_4_ = 3.711, *p* = 0.021 for vehicle). In light of previous findings on object-recognition impairment mediated by aberrant DA release in *Fmr1*-KO mice [48], our results indicate intranasal application of DA is an effective avenue for ameliorating the cognitive deficits in the FXS model.

In the three-chamber social test, no group differences were noticed in general explorative behaviors in any of the sessions (*p* > 0.05; Table 2). In the sociability trial, there was a significant effect of “object” (F_1,13_ = 73.735, *p* < 0.001), but not of “group” or their interaction (*p* > 0.05). Both vehicle and DA groups explored the stranger mouse more than the empty cup (t_6_ = 5.85, *p* = 0.001; t_7_ = 6.493, *p* < 0.001, respectively) with equally positive sociability indexes (t_6_ = 7.571, *p* < 0.001; t_7_ = 12.666, *p* < 0.001, respectively; Fig. 6c). In the social novelty trial, significant effects of “object” (F_1,13_ = 9.375, *p* = 0.009) and “group x object” (F_1,13_ = 11.313, *p* = 0.005), but not of “group” (*p* > 0.05), were found. The DA-treated animals explored the novel stranger more than the familiar one (t_7_ = − 3.756, *p* = 0.007), but the vehicle-treated group did not (*p* > 0.05). Thereby, the DA treatment elevated the social novelty index (t_7_ = − 4.45, *p* = 0.003), however the vehicle failed to do so (*p* > 0.05; Fig. 6c). Knowing that *Fmr1*-KO animals have normal social approaching but atypical social novelty preference [33, 49], we suggest that intranasal DA particularly alleviates the impaired social novelty in the autistic-like *Fmr1*-KO model.

In the elevated plus maze test, there were no group differences in the behavioral measurements (*p >* 0.05; Table 3), indicating a minimal effect of DA on the anxiety level of *Fmr1*-KO mice.

## Discussion

In this comparative study, we have unraveled distinct alterations and common phenotypes in the DA pathways of two widely adopted mouse models for ASD. BTBR mice showed a hypofunction of the DA system, as indicated by the low expression of TH in several DA centers (Figs. 1, 2, 3), in line with previous studies showing compromised DA-mediated responses in these mice [20]. Moreover, they exhibited decreased motivation for social and food rewards in operant conditioning tasks [50] and less social conditioned place preference [51], which reinforces the perspective of dysfunctional DA system in this model. As TH is an enzyme for synthesis of both DA and norepinephrine, future studies are required to differentiate their roles in the BTBR brain. For *Fmr1*-KO mice, the TH level did not change significantly (Fig. 3), largely consistent with other reports [23, 24, 49]. Yet, fractal analysis revealed unusual arborization of TH-positive axons in their STR (Fig. 2), strengthening an essential role of FMRP in axon formation [52]. For instance, a loss of FMRP homologue dFMR1 in *Drosophila* generated aberrant extensions and branches of axons [53, 54], while overexpression of dFMR1 led to abridged axonal arbors [55]. In cultured rat cortical neurons, FMRP overexpression attenuated the axon complexity [56]. Furthermore, the axon integrity was altered in the cortex of *Fmr1-*KO mice [57] and in the dSTR of FXS patients [58]. Despite of the controversy [59], FMRP also plays a role in the development of axon myelination [60, 61]. Whether defective myelination could contribute to the abnormal morphology of TH-positive axons in the *Fmr1*-KO STR remains unclear. The differences in TH expression between BTBR and *Fmr1*-KO mice could relate to their individual genetic background as well.

Co-labeling VGLUT1 with TH showed an increased number of VGLUT1-containing nerve terminals in close spatial relationship with TH-positive axons, indicating an enhanced interaction between the cortical afferents and released DA in the STR of the two ASD mouse lines (Fig. 2). The molecular underpinnings of DA modulation are complex, for example, depending on the subtypes of DA receptors [62]. Whether DA facilitates or attenuates glutamatergic neurotransmission will be subject to further investigations. Another commonality between the two models was the downregulation of striatal DAT (Fig. 3). DAT is critical for maintaining DA homeostasis by recycling DA from the synaptic cleft to the cytosol. Whole-exome sequencing has identified a DAT mutation in ASD families [8]. Transgenic mice with DAT deficiency showed hyperactivity [63]. Administration of amphetamine, which causes DAT-mediated DA efflux, alleviated self-grooming in BTBR mice [64] and facilitated object recognition in *Fmr1*-KO mice [48]. More studies are needed to elucidate the mechanisms and consequences of the DAT defect.

The causes for the protein regulation by intranasal DA in the ASD models may be diverse. The bidirectional modulation of the quantity of TH in the BTBR and *Fmr1*-KO brain (Fig. 4) likely depends on the different changes in their endogenous DA system (Fig. 3). DA administration in BTBR mice may increase the extracellular DA concentration and DA availability [65], which presumably increases the TH activity in the STR [66]. In *Fmr1*-KO mice, a lack of FMRP may play a role in reducing the level of TH, considering the interaction between FMRP and DA signaling [25, 67]. This possibility is supported by an observation that intranasal DA did not change TH protein in normal rats [68]. As to DAT, increased extracellular DA could affect its binding activity [69]. In spite of the evidence for striatal DAT deficiency in the two strains (Fig. 3), intranasal delivery of DA did not restore DAT expression (Fig. 4).

Intranasal application of DA efficiently rescued the cognitive and social deficits of the BTBR (Fig. 5) and *Fmr1*-KO (Fig. 6) models. It should be noted that the behavioral assessments of these functions can be confounded by other factors. For instance, hyper- or hypo-locomotor activity may influence animals’ performance in the object-based attention test and three-chamber social test. However, this unlikely compromised the effects of DA on cognition and social interaction as the overall motor and exploratory behaviors were comparable between the DA and vehicle groups (Figs. 5 & 6). In rats treated with intranasal DA, an elevated concentration of DA was found in the cerebrospinal fluid and in the brain [70], including the dSTR and NAc [65]. Moreover, intranasal DA had antidepressant-like effects [71], attenuated fear responses [72], compensated behavioral asymmetries in a Parkinsonism model [73], and alleviated cognitive deficits in aged rats [74] as well as in animal models for schizophrenia [75] and ADHD [76]. Therefore, the behavioral rescues by intranasal DA likely stem from brain-wide actions. Here, we focused on the STR because it is a key neuronal correlate to social behaviors [77] and a disparate fronto-striatal circuit has been specified in ASD patients [78, 79]. Mice with deletion of ASD-relevant genes, e.g., *Shank3*, displayed social impairments, along with decreased corticostriatal neurotransmission, increased morphological complexity of medium spiny neurons, and reduced glutamate receptors in the STR [77]. Although we (Fig. 3) and others did not note dramatic changes in TH expression in *Fmr1*-KO animals [23, 24], DA release and uptake were blunted in their STR [24]. The syndromic ASD model, BTBR mice with polygenetic mutations [27], presented severe detriments in production and reuptake of DA as manifested by reduced striatal TH and DAT proteins, respectively (Fig. 3). These findings suggest that the defective STR and its connected circuitry are major features of ASD. Furthermore, the susceptibility of striatal TH protein to intranasal DA treatment in BTBR and *Fmr1*-KO mice (Fig. 4) indicate that the STR can be an effective target for therapeutic interventions of ASD. Our results provide not only empirical evidence for the DA hypothesis of ASD [80], but also a proof of principle for developing clinical treatments for the disorder.

## Data Availability

All the data are available upon request.

## Abbreviations

ASD: Autistic spectrum disorder
FXS: Fragile X syndrome
ADHD: Attention-deficit/hyperactivity disorder
BTBR: Black and Tan BRachyury T^+^Itpr3^tf^/J
*Fmr1*-KO: Fragile X Mental Retardation 1 knockout
WT: Wild type
DA: Dopamine
TH: Tyrosine hydroxylase
DAT: Dopamine transporter
VGLUT1: Vesicular glutamate transporter 1
GAD67: Glutamic acid decarboxylase 67
SNc: Substantia nigra pars compacta
VTA: Ventral tegmental area
STR: Striatum
dSTR: Dorsal striatum
NAc: Nucleus accumbens
ROI: Region of interest
Db: Box-counting dimension
SEM: Standard error of mean
